# Supplementary material for: Visceral obesity and insulin resistance associate with CD36 deletion in lymphatic endothelial cells
Source: Nat Commun. 2021 Jun 7;12:3350. doi: 10.1038/s41467-021-23808-3 (PMC8184948; doi:10.1038/s41467-021-23808-3)
Supplement: Supplementary file 1 — Supplementary Information [file 41467_2021_23808_MOESM1_ESM.pdf]

Supplementary Information

Visceral obesity and insulin resistance associate with CD36 deletion in lymphatic endothelial cells.

Cifarelli et al.

SUPPLEMENTARY METHODS

Mesentery whole-mount imaging

Mesentery was harvested and fixed overnight in 1% paraformaldehyde. The next day, tissue was washed with PBS three times 10 minutes each, permeabilized with PBS + 0.3% Triton X-100 (PBST) for one hour, and blocked with 3% donkey serum in PBST for two hours. Primary antibodies were mixed in PBST, added to the tissue, and incubated overnight. To remove primary antibodies, tissue was washed five times 15 minutes each with PBST. Secondary and conjugated antibodies were mixed in PBST, added to the tissue, and incubated at room temperature for two hours on an orbital shaker. Tissue was then washed five times 15 minutes each with PBST. To provide nuclear contrast, DAPI was dissolved in PBS, added to the tissue, and incubated at room temperature for 5 minutes, followed by a wash in PBS for 5 minutes. Mesentery were then mounted on glass slides (Superfrost Plus Microscope slides, Fisherbrand) with ProLong Diamond Antifade Mountant containing DAPI (Invitrogen) and stored at 4°C overnight before imaging on a Zeiss LSM 880 Airyscan Confocal Microscope.

Plasma Triglycerides

Fasted (12 h) mice received an i.p. injection of Triton WR-1339 (200 mg per kg body weight) (Sigma) 30 min before olive oil (10 µl/g of body weight) gavage with subsequent measurement of TG concentrations. Tail blood samples were collected before and 1, 3, 5 and 7 h after bolus administration. Plasma TG was assayed with an Infinity Kit (ThermoFisher Scientific).

Plasma BODIPY C16

Fasted (12h) mice received 0.1 mg/kg of BODIPY FL C_{16} (4,4-difluoro-5,7-dimethyl-4-bora-3a, 4a-diaza-s-indacene-3-hexadecanoic acid, Molecular Probes, Inc.) with 0.2% fatty-free bovine serum albumin (BSA) by gavage. Tail blood samples were collected at 0, 60 and 120 min and

fluorescence intensity was measured (λex = 488/λem = 515 nm) in black, 96-well flat-bottom plates using a fluorescence microplate reader. The relative fluorescence units (RFU) signal from BODIPY-treated mice was subtracted from the one measured in mice receiving control solution (BSA only).

Lymphatic endothelial cell flow cytometry

Human Dermal Lymphatic Endothelial Cells (LECs, PromoCell) were cultured in EBM-2MV medium (Lonza). Serum-starved LECs (6h) were treated with/without 100 ng/ml VEGF-C (R&D System) for 20 min at 37°C, collected using cell dissociation buffer (enzyme-free) (ThermoFisher) and stained (Supplementary Table 1) for 20 min on ice. Doublets and dead cells, positive for LIVE/DEAD Fixable Aqua (Life Technologies), were excluded from analyses. Cells were analyzed using FACS Fortessa (BD Biosciences) and FlowJo v10 software (Treestar).

SUPPLEMENTARY FIGURES

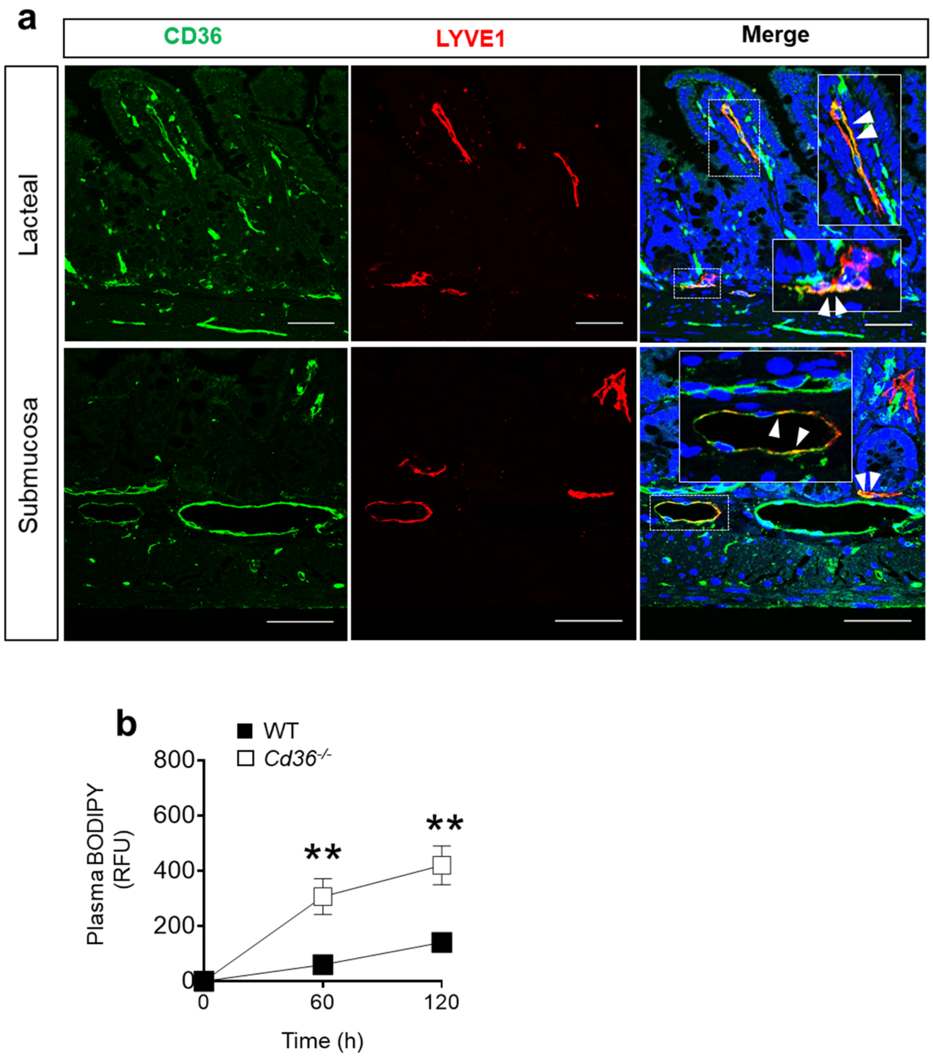

Supplementary Fig. 1: Expression of CD36 in gut lymphatics. a Representative images of paraffin-embedded intestinal sections showing individual staining and co-localization for CD36 (green) and lymphatic vessel endothelial hyaluronan receptor 1, LYVE-1 (red) by immunofluorescence in lymphatic vessels of the small intestine (jejunum) in C57BL/6 mice (*n* = 5). Scale bar is 50 µm. Images are representative of two independent experiments. **b** Fluorescent long-chain fatty acid BODIPY C16 appearance in blood in 14-week-old wildtype, WT and *Cd36*$^{-/-}$ mice (*n* = 4) over 120 minutes (60 min and 120 min, both $P<0.001$). All data are from two independent experiments with *n* representing the number of mice per group. RFU: Relative fluorescent unit. Data are means ± SE. Statistical significance is determined by two-tailed Student *t* test. **, $P<0.01$.

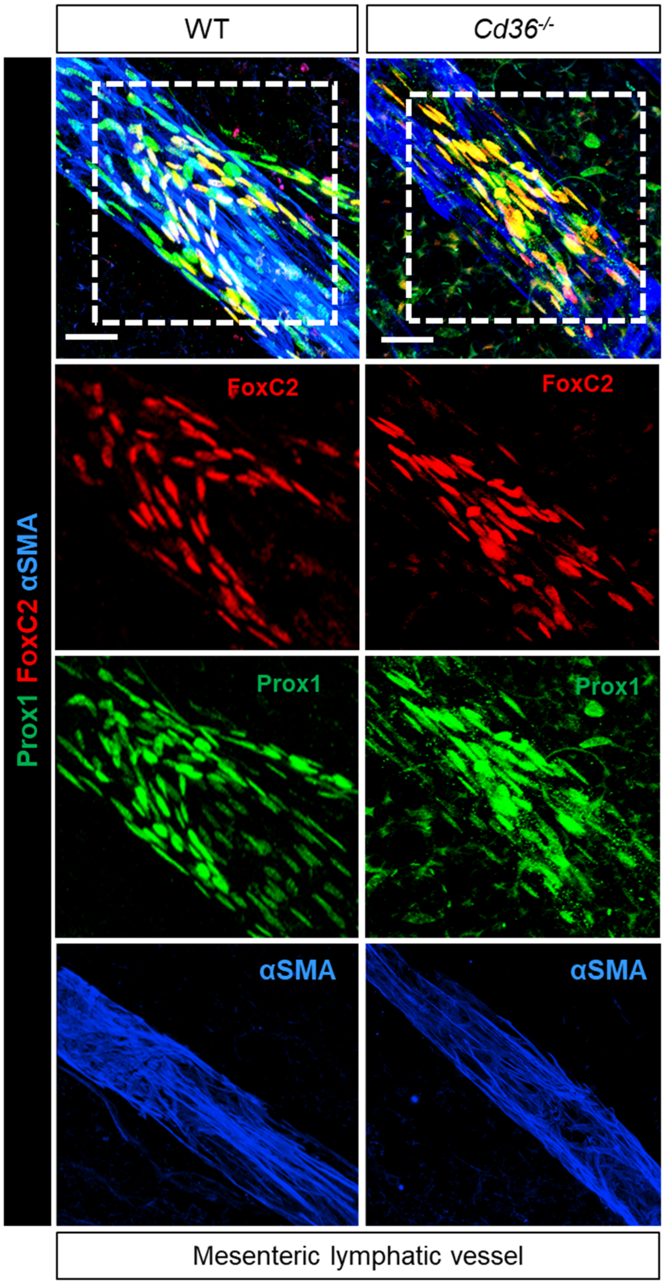

Supplementary Fig. 2: CD36 deletion does not affect valve morphology in mesentery collecting lymphatics. Representative images of whole-mount mesentery lymphatic vessels showing co-localization and individual staining for Forkhead box protein C2, FoxC2 (red), Prospero homeobox protein 1, Prox1 (green), and Smooth muscle actin-α, SMAα (blue) in 12-week-old wildtype (WT) and *Cd36*$^{-/-}$ mice (*n* = 3). Scale bar: 50 μm. Images are representative of two independent experiments with *n* representing the number of mice per group.

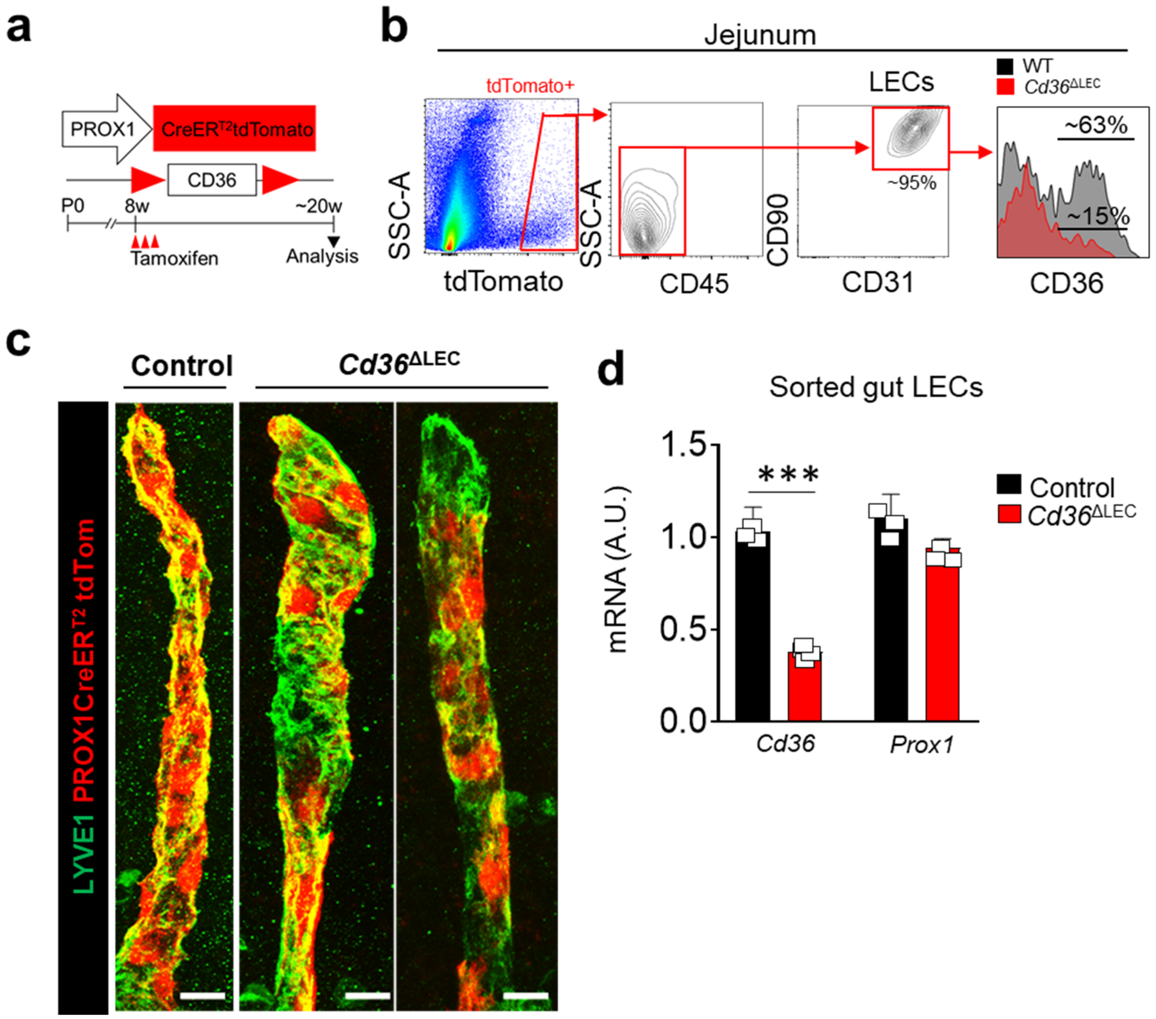

Supplementary Fig. 3: Generation of mice with inducible *Cd36* deletion in lymphatic endothelial cells, LECs. a *Cd36*$^{fl/fl}$ mice are crossed with Prox1-CreER^{T2}-tdTomato mice and *Cd36* deletion in Prox1-CreER^{T2}-tdTomatoCd36$^{fl/fl}$ (*Cd36*$^{\Delta LEC}$) induced by intragastric administration of tamoxifen at 8-week of age. Controls are Flox negative Cre positive littermate mice that receive the same tamoxifen protocol. **b** TdTomato^{+} cells are mostly (~95%) LECs (CD45^{-}CD90.2^{+}CD31^{+}). **c** Whole-mount staining of Control and *Cd36*$^{\Delta LEC}$ jejunums showing tdTomato signal localizes with LYVE-1 (green fluorescence). Images are acquired using a Zeiss LSM 880 Airyscan Confocal Microscope, 63X objective. Scale bar: 5 µm. Images are representative of three independent experiments. **d** mRNA expression of *Cd36* (*P*<0.001) and *Prox1* in sorted intestinal tdTomato^{+} LECs from Control and *Cd36*$^{\Delta LEC}$ mice by qRT-PCR. Data are means ± SE from *n* = 4 mice per group from two independent experiments. A.U., Arbitrary unit. SSC, Side scatter. CD, Cluster of differentiation. Statistical significance is determined by two-tailed Student *t* test. ***, *P*<0.01.

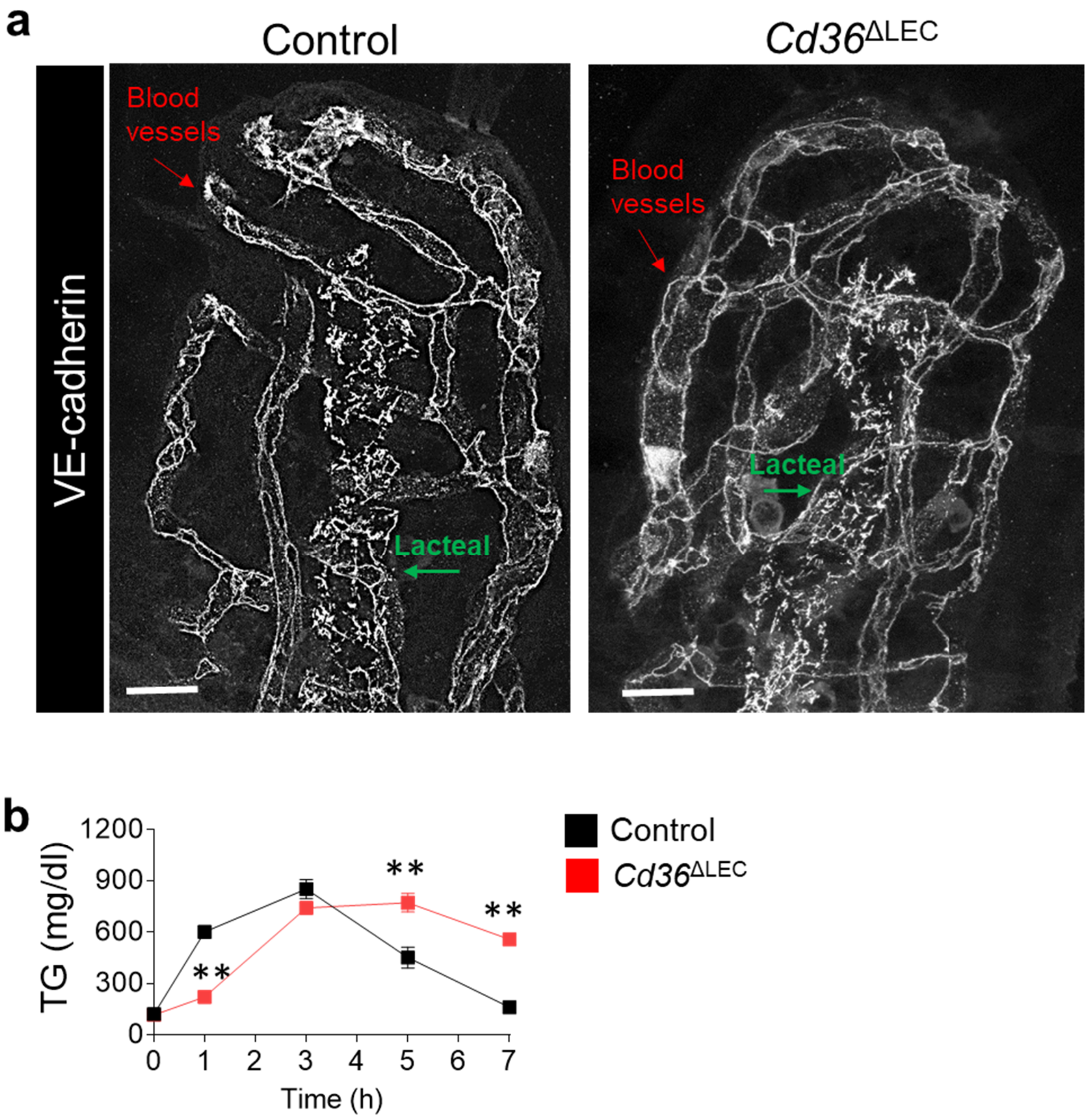

Supplementary Fig. 4: VE-cadherin staining in villi of Control and *Cd36*$^{\Delta LEC}$ mice. a Representative images of whole-mount jejunum showing VE-cadherin immunostaining in villi of control and *Cd36*$^{\Delta LEC}$ mice (n = 4). Images are acquired in tiled scans using a Zeiss LSM 880 Airyscan Confocal Microscope, objective: 40X. Scale bar: 100 µm. (b) Plasma triglyceride (TG) levels after intragastric administration of olive oil (10µl/g of body weight) to 12 h fasted mice (n = 4/group) measured over 0-7 h. Plasma TG levels in *Cd36*$^{\Delta LEC}$ mice were reduced at 1 h (P<0.01), maintained a plateau starting at 3h, as levels in controls decrease (5 and 7 h points P<0.01). All data are means ± SE. Statistical significance is determined by two-tailed Student t test. **, P<0.01.

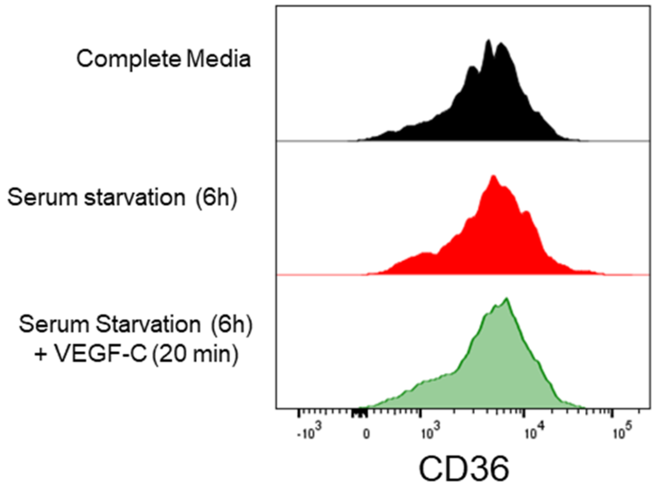

Supplementary Fig. 5: CD36 expression by flow cytometry in human dermal lymphatic endothelial cells (LECs). Surface CD36 expression by flow cytometry on LECs did not change following serum starvation (6 h) and/or VEGF-C treatment (20 min) as compared with LECs cultured in complete media only. Histogram is representative of three experiments.

Supplementary Table 1. List of antibodies

Immunofluorescence					
**Antibody**	**Species**	**Identifier**	**Cat. No.**	**Clone**	**Dilution**
LYVE-1	Rabbit anti-mouse	AngioBio	11-034	polyclonal	1:500
Alexa-Fluor^{TM} 647 VE-cadherin	Rat anti-mouse	BD Bioscience	562242	11D4.1	1:50
VE-cadherin	Goat anti-human	SCBT	Sc-6458	polyclonal	1:200
Cy3^{TM} SMA	Mouse anti-SMA	Sigma	C6198	clone 1A4	1:200
CD36	Goat anti-mouse	R&D System	AF2519	polyclonal	1:200
Foxc2	Sheep anti-mouse	R&D System	AF6889	polyclonal	1:200
Prox1	Rabbit anti-mouse	Origene	DP3516	polyclonal	1:500
**Flow Cytometry**					
CD45-FITC	Rat anti-mouse	Biolegend	103107	30-F11	1:200
CD31-PeCy7	Rat anti-mouse	Biolegend	102417	390	1:800
CD90.2-PerCPCy5.5	Rat anti-mouse	Biolegend	105338	30-H12	1:100
CD36-APC	Hamster anti-mouse	Biolegend	102612	HM36	1:100
IgG Isotype Ctrl	Armenian Hamster	Biolegend	400911	HTK888	1:100
CD36-PE	Mouse anti-Human	Biolegend	305105	G10F5	1:100
**Western Blotting**					
VE-cadherin	Rabbit anti-human	Abcam	Ab33168	polyclonal	1:1000
CD36	Goat anti-human	R&D System	AF1955	polyclonal	1:500
AKT	Rabbit anti-human	Cell Signaling	9272	polyclonal	1:1000
p-AKT^{S473}	Rabbit anti-human	Cell Signaling	4060	D9E	1:1000
VEGFR2	Rabbit anti-human	Cell Signaling	2479	55B11	1:1000
p-VEGFR2^{Tyr1175}	Rabbit anti-human	Cell Signaling	3770	D5B11	1:1000
β-Actin	Mouse anti-human	SCBT	sc-47778	C4	1:1000

Supplementary Table 2. List of primer sequences

Primers	Forward	Reverse
**mVegfc**	5’-AACCTCCATGTGTGTCCGTC-3’	5’-CAAACAACGTCTTGCTGAGGT-3’
**mDll4**	5’-TGCCACTTCGGTTACACAGT-3’	5’-AGGCTCCTGCCTTATACCTCT-3’
**mCD36 exon 3/4**	5’-GTTATTGGTGCAGTCCTGGC-3’	5’-GGTTCCTTCTTCAAG GACAACTT-3’
**mCD36**	5’-GATGACGTGGCAAAGAACAG-3’	5’-CAGTGAAGGCTCAAAGATGG-3’
**mTnf**	5’-CACCACGCTCTTCTGTCTACTG-3’	5’-CTTTGAGATCCATCGCGTTG-3’
**mIl6**	5’-AGCCAGAGTCCTTCAGAGAGAT-3’	5’-GCACTAGGTTTGCCGAGTAGAT-3’
**mProx1**	5’-GCTACCCCAGCTCCAACATGCT-3’	5’-TGATGGCTTGACGCGCATACTTCT-3’
**mCpt1a**	5’-GCCAGGAGGTCATAGAGACAT-3’	5’-GAGTCATGGAAGCCTCATACG-3’
**mEmr1**	5’-CCTGATGGTGAGAAACCTGA-3’	5’-CCCCAGGAAACTCCAGATAA-3’
**mPparg**	5’-GCCCTTTGGTGACTTTATGG-3’	5’-GTCATCTTCTGGAGCACCTTG-3’
**mTgfb1**	5’-GTGGACCGCAACAACGCCATC-3’	5’-TGGGGGTCAGCAGCCGGTTA-3’
**mLpl**	5’-GAAACCAGTAGGGCATGTTG-3’	5’-CATTCAGCAGGGAGTCAATG-3’
**m36b4**	5’-GCAGACAACGTGGGCTCCAAGCAGAT-3’	5’-GGTCCTCCTTGGTGAA-3’
**hCpt1a**	5’-GCCAGGAGGTCATAGAGACAT-3’	5’-GAGTCATGGAAGCCTCATACG-3’
**hAcsl1**	5’-GGAAGAGCCAACAGACGGAA-3’	5’-CCTTTGGGGTTG CCTGTAGT-3’
**hVlcad**	5’-CGGAGAGATTCGGAGATGCA-3’	5’-CCACCATCTCCAGAGCGTCATT-3’
**hSlc27a1**	5’-CCACTTGGATGTCACCACTG-3’	5’-GTGGGACCCTCCAGTAGACA-3’
**hSlc27a3**	5’-ATACCTGGGAGCGTTTTGTG-3’	5’-CCGCTGTCCTGTGTAGTTGA-3’
**hSlc27a4**	5’-GCTTCGATGGCTACCTCAAC-3’	5’-GTCCATCACCAGCACCATAC-3’
**hHk2**	5’-GAGTTTGACCTGGATGTGGTTGC-3’	5’-CCTCCATGTAGCAGGCATTGCT-3’
**hPfkfb3**	5’-GGCAGGAGAATGTGCTGGTCAT-3’	5’-CATAAGCGACAGGCGTCAGTTTC-3’
**hAldoa**	5’-GACACTCTACCAGAAGGCGGAT-3’	5’-GGTGGTAGTCTCGCCATTTGTC-3’
**hSlc2a1 (GLUT1)**	5’-TTGCAGGCTTCTCCAACTGGAC-3’	5’-CAGAACCAGGAGCACAGTGAAG-3’
**h36B4**	5’-GTGATGTGCAGCTGATCAAGACT-3’	5’-GATGACCAGCCCAAAGGAGA-3’
