# Supplementary material for: Visceral obesity and insulin resistance associate with CD36 deletion in lymphatic endothelial cells
Source: Nat Commun. 2021 Jun 7;12:3350. doi: 10.1038/s41467-021-23808-3 (PMC8184948; doi:10.1038/s41467-021-23808-3)
Supplement: Supplementary file 3 — Reporting Summary [file 41467_2021_23808_MOESM3_ESM.pdf]

nature research

Corresponding author(s): *Vincenza Cifarelli & Nada Abumrad*

Last updated by author(s): *2021-04-27*

Reporting Summary

Nature Research wishes to improve the reproducibility of the work that we publish. This form provides structure for consistency and transparency in reporting. For further information on Nature Research policies, see our Editorial Policies and the Editorial Policy Checklist.

Please do not complete any field with "not applicable" or n/a. Refer to the help text for what text to use if an item is not relevant to your study.
For final submission: please carefully check your responses for accuracy; you will not be able to make changes later.

Statistics

For all statistical analyses, confirm that the following items are present in the figure legend, table legend, main text, or Methods section.

n/a	Confirmed	
[ ]	[X]	The exact sample size (*n*) for each experimental group/condition, given as a discrete number and unit of measurement
[X]	[ ]	A statement on whether measurements were taken from distinct samples or whether the same sample was measured repeatedly
[ ]	[X]	The statistical test(s) used AND whether they are one- or two-sided<br>*Only common tests should be described solely by name; describe more complex techniques in the Methods section.*
[X]	[ ]	A description of all covariates tested
[X]	[ ]	A description of any assumptions or corrections, such as tests of normality and adjustment for multiple comparisons
[ ]	[X]	A full description of the statistical parameters including central tendency (e.g. means) or other basic estimates (e.g. regression coefficient) AND variation (e.g. standard deviation) or associated estimates of uncertainty (e.g. confidence intervals)
[ ]	[X]	For null hypothesis testing, the test statistic (e.g. *F*, *t*, *r*) with confidence intervals, effect sizes, degrees of freedom and *P* value noted<br>*Give P values as exact values whenever suitable.*
[X]	[ ]	For Bayesian analysis, information on the choice of priors and Markov chain Monte Carlo settings
[X]	[ ]	For hierarchical and complex designs, identification of the appropriate level for tests and full reporting of outcomes
[X]	[ ]	Estimates of effect sizes (e.g. Cohen's *d*, Pearson's *r*), indicating how they were calculated

Our web collection on statistics for biologists contains articles on many of the points above.

Software and code

Policy information about availability of computer code

Data collection	LSR Fortessa and FACSAria II (bothBD Bioscience) was used to collect flow cytometry data.
Data analysis	FlowJo_VI0 was used for flow cytometry data analysis. Image Studio Lite v5.2 was used for band quantification obtained by western blotting . NIH ImageJ v1.53i was used for microscopy data. All the plots were generated by Graph Pad Prism (Version 8.4.3)

For manuscripts utilizing custom algorithms or software that are central to the research but not yet described in published literature, software must be made available to editors and reviewers. We strongly encourage code deposition in a community repository (e.g. GitHub). See the Nature Research guidelines for submitting code & software for further information.

Data

Policy information about availability of data

All manuscripts must include a data availability statement. This statement should provide the following information, where applicable:
- Accession codes, unique identifiers, or web links for publicly available datasets
- A list of figures that have associated raw data
- A description of any restrictions on data availability

The uncropped immunoblots used in this study are provided in the Source Data. Source data are provided with this paper. Data are available and can be obtained from the corresponding authors upon resonable request.

Field-specific reporting

Please select the one below that is the best fit for your research. If you are not sure, read the appropriate sections before making your selection.

[X] Life sciences [ ] Behavioural & social sciences [ ] Ecological, evolutionary & environmental sciences

For a reference copy of the document with all sections, see nature.com/documents/nr-reporting-summary-flat.pdf

Life sciences study design

All studies must disclose on these points even when the disclosure is negative.

Sample size	No sample size calculations were performed. Sample size was determined according to preivous publications (PMID:28066800;PMID:30047927;)
Data exclusions	No samples or animals were excluded from the analysis.
Replication	At least 3 independent repeats were included for related experiments. Each experiment was performed for at least twice to make sure similar results are reproducible. All attempts of replications were succesful
Randomization	Sex- and age-matched mice models or samples were used in all experiments.
Blinding	Cells receiving Ctrl or CD36 siRNA were randomly assigned. For cell-based experiments, western blotting, FACS and animal experiments, cell treatments were known in order to prepare the samples or start treatment at the beginning of experiments.

Behavioural & social sciences study design

All studies must disclose on these points even when the disclosure is negative.

Study description	N/A
Research sample	N/A
Sampling strategy	N/A
Data collection	N/A
Timing	N/A
Data exclusions	N/A
Non-participation	N/A
Randomization	N/A

Ecological, evolutionary & environmental sciences study design

All studies must disclose on these points even when the disclosure is negative.

Study description	N/A
Research sample	N/A

Research sample	N/A
Sampling strategy	N/A
Data collection	N/A
Timing and spatial scale	N/A
Data exclusions	N/A
Reproducibility	N/A
Randomization	N/A
Blinding	N/A

Did the study involve field work? ☐ Yes ☒ No

Field work, collection and transport

Field conditions	N/A
Location	N/A
Access & import/export	N/A
Disturbance	N/A

Reporting for specific materials, systems and methods

We require information from authors about some types of materials, experimental systems and methods used in many studies. Here, indicate whether each material, system or method listed is relevant to your study. If you are not sure if a list item applies to your research, read the appropriate section before selecting a response.

Materials & experimental systems

n/a	Involved in the study	
x	X	Antibodies
	X	Eukaryotic cell lines
x		Palaeontology and archaeology
	X	Animals and other organisms
x		Human research participants
x		Clinical data
x		Dual use research of concern

Methods

n/a	Involved in the study	
x		ChIP-seq
	X	Flow cytometry
x		MRI-based neuroimaging

Antibodies

Antibodies used	*All antibodies, supplier name, clones and catalog numbers used in this study are provided in Supplemental Table 1*
Validation	All antibodies used in our study have been validated and detailed information could be found on the website from manufactures (AngioBio, BD Bioscience, SCBT, R&D System, Biolegend, Cell Signaling). APC anti-mouse CD36 antibody (clone HM36, Biolegend cat.# 102812) for flow cytometry as reported by others (De Silva N, 2016, PNAS,PMID: 27457956;Misumi I,2019, Cell Rep,PMID: 30970254). We tested the CD36 Ab in Cd36 null (Cd36-/-) LECs and observed no signal (Fig.1c).

Eukaryotic cell lines

Policy information about cell lines

Cell line source(s)	Primary Human Dermal Lymphatic Endothelial Cells (HDLECs) were obtained from PromoCells (cat # 12216)

Authentication | Authenticated was provided by the Manufacturer (PromoCell) by podoplanin expression. Cells were not further authenticated in our laboratory.

Mycoplasma contamination | Cell line tested negative for mycoplasma contamination.

Commonly misidentified lines (See ICLAC register) | No ICLAC cell line was used in this study

Palaeontology and Archaeology

Specimen provenance | N/A

Specimen deposition | N/A

Dating methods | N/A

☐ Tick this box to confirm that the raw and calibrated dates are available in the paper or in Supplementary Information.

Ethics oversight | N/A

Note that full information on the approval of the study protocol must also be provided in the manuscript.

Animals and other organisms

Policy information about studies involving animals; ARRIVE guidelines recommended for reporting animal research

Laboratory animals | Male and female WT and CD36-/- mice, both on C57BL/6 strain, were used at 12-14 week of age. Cd36deltaLEC and controls, both on a C57BL/6 strain, were used at 11- and 20-week old of age. All mice were maintained at a condition of 12-hour light/12-hour dark cycle and temperatures of 65-75°F (18-23°C) with 40-60% humidity.

Wild animals | No wild animals were involved in this study.

Field-collected samples | This study didn't involve samples collected from field.

Ethics oversight | All the mouse experiments were performed in accordance with a protocol approved by the Institutional Animal Care and Use Committee and Institutional Review Board at Washington University School of Medicine

Note that full information on the approval of the study protocol must also be provided in the manuscript.

Human research participants

Policy information about studies involving human research participants

Population characteristics | N/A

Recruitment | N/A

Ethics oversight | N/A

Note that full information on the approval of the study protocol must also be provided in the manuscript.

Clinical data

Policy information about clinical studies

All manuscripts should comply with the ICMJE guidelines for publication of clinical research and a completed CONSORT checklist must be included with all submissions.

Clinical trial registration | N/A

Study protocol | N/A

Data collection | N/A

Outcomes | N/A

Dual use research of concern

Policy information about dual use research of concern

Hazards

Could the accidental, deliberate or reckless misuse of agents or technologies generated in the work, or the application of information presented in the manuscript, pose a threat to:

No	Yes	
X	☐	Public health
X	☐	National security
X	☐	Crops and/or livestock
X	☐	Ecosystems
X	☐	Any other significant area

Other impacts

Hazards

For examples of agents subject to oversight, see the United States Government Policy for Institutional Oversight of Life Sciences Dual Use Research of Concern.

Experiments of concern

Does the work involve any of these experiments of concern:

No	Yes	
X	☐	Demonstrate how to render a vaccine ineffective
X	☐	Confer resistance to therapeutically useful antibiotics or antiviral agents
X	☐	Enhance the virulence of a pathogen or render a nonpathogen virulent
X	☐	Increase transmissibility of a pathogen
X	☐	Alter the host range of a pathogen
X	☐	Enable evasion of diagnostic/detection modalities
X	☐	Enable the weaponization of a biological agent or toxin
X	☐	Any other potentially harmful combination of experiments and agents

Other combinations: *Describe any other potentially harmful combination(s) of experiments and agents.*

Precautions and benefits

Biosecurity precautions: N/A

Biosecurity oversight: N/A

Benefits: N/A

Communication benefits: N/A

ChIP-seq

Data deposition

☐ Confirm that both raw and final processed data have been deposited in a public database such as GEO.

☐ Confirm that you have deposited or provided access to graph files (e.g. BED files) for the called peaks.

Data access links
May remain private before publication.
N/A

Files in database submission: N/A

Genome browser session (e.g. UCSC): N/A

Methodology

Replicates: N/A

Sequencing depth: N/A

Antibodies: N/A

Peak calling parameters: N/A

Data quality: N/A

Software: N/A

Flow Cytometry

Plots

Confirm that:

- [X] The axis labels state the marker and fluorochrome used (e.g. CD4-FITC).
- [X] The axis scales are clearly visible. Include numbers along axes only for bottom left plot of group (a 'group' is an analysis of identical markers).
- [X] All plots are contour plots with outliers or pseudocolor plots.
- [X] A numerical value for number of cells or percentage (with statistics) is provided.

Methodology

Sample preparation: Intestinal and mesentery cell suspensions were prepared usingthe Miltenyi Lamina Propria Dissociation kit (Miltenyi Biotech). Intestines were separated off the mesentery. The jejunum was then opened longitudinally and, after removal of Peyer’s patches, gently scraped off to physically separate the mucosal layer(lacteals) from the submucosaby using a cell scraper. The submucosa was washed twice with DTT/EDTA on a shaker at 37°C, followed by enzymatic digestion for 30 min, whereas gut mucosa and mesentery were directly digested for 30 min. The three cell suspensions were stained with an antibody cocktail (Supplementary Table 1)

Instrument: BD LSR Fortessa and FACSAria II were used to collect flow cytometry data.

Software: Cells were acquired using BD FACS DIVA and data analysed conducted by using FlowJo software (Treestar).

Cell population abundance: At least 10,000 cells (LECs) were isolated from the intestine of Cd36DeltaLEC and control mice. Purity was determining by checking expression of CD31+CD90+ by flow cytometry and of key lymphangiogenic genes by qRT-PCR.

Gating strategy: Gating strategy is presented in Figure 1b
We utilized FSC/SSC; CD45-CD31+CD90+; CD36+ as previuosly published by Ogasawara 2018 PMID 30013036.

- [X] Tick this box to confirm that a figure exemplifying the gating strategy is provided in the Supplementary Information.

Magnetic resonance imaging

Experimental design

Design type: N/A

Design specifications: N/A

Behavioral performance measures: N/A

Acquisition

Imaging type(s)	N/A
Field strength	N/A
Sequence & imaging parameters	N/A
Area of acquisition	N/A

Diffusion MRI ☐ Used ☐ Not used

Parameters

Preprocessing

Preprocessing software	N/A
Normalization	N/A
Normalization template	N/A
Noise and artifact removal	N/A
Volume censoring	N/A

Statistical modeling & inference

Model type and settings	N/A
Effect(s) tested	N/A

Specify type of analysis: ☐ Whole brain ☐ ROI-based ☐ Both

Anatomical location(s) N/A

Statistic type for inference (See Eklund et al. 2016)	N/A
Correction	N/A

Models & analysis

n/a	Involved in the study
X	☐ Functional and/or effective connectivity
X	☐ Graph analysis
X	☐ Multivariate modeling or predictive analysis

Functional and/or effective connectivity	N/A
Graph analysis	N/A
Multivariate modeling and predictive analysis	N/A

This checklist template is licensed under a Creative Commons Attribution 4.0 International License, which permits use, sharing, adaptation, distribution and reproduction in any medium or format, as long as you give appropriate credit to the original author(s) and the source, provide a link to the Creative Commons license, and indicate if changes were made. The images or other third party material in this article are included in the article's Creative Commons license, unless indicated otherwise in a credit line to the material. If material is not included in the article's Creative Commons license and your intended use is not permitted by statutory regulation or exceeds the permitted use, you will need to obtain permission directly from the copyright holder. To view a copy of this license, visit http://creativecommons.org/licenses/by/4.0/
